# A robust gene expression signature for NASH in liver expression data

**DOI:** 10.1038/s41598-022-06512-0

**Published:** 2022-02-16

**Authors:** Yehudit Hasin-Brumshtein, Suraj Sakaram, Purvesh Khatri, Yudong D. He, Timothy E. Sweeney

**Affiliations:** 1 Inflammatix, Inc., 863 Mitten Rd, Suite 104, Burlingame, CA 94010 USA; 2 Institute for Immunity, Transplantation and Infection, School of Medicine, Stanford University, Palo Alto, CA 94305 USA; 3 Department of Medicine, Center for Biomedical Informatics Research, Stanford University, Stanford, CA 94305 USA

**Keywords:** Diagnostic markers, Non-alcoholic fatty liver disease, Non-alcoholic steatohepatitis

## Abstract

Non-Alcoholic Fatty Liver Disease (NAFLD) is a progressive liver disease that affects up to 30% of worldwide population, of which up to 25% progress to Non-Alcoholic SteatoHepatitis (NASH), a severe form of the disease that involves inflammation and predisposes the patient to liver cirrhosis. Despite its epidemic proportions, there is no reliable diagnostics that generalizes to global patient population for distinguishing NASH from NAFLD. We performed a comprehensive multicohort analysis of publicly available transcriptome data of liver biopsies from Healthy Controls (HC), NAFLD and NASH patients. Altogether we analyzed 812 samples from 12 different datasets across 7 countries, encompassing real world patient heterogeneity. We used 7 datasets for discovery and 5 datasets were held-out for independent validation. Altogether we identified 130 genes significantly differentially expressed in NASH versus a mixed group of NAFLD and HC. We show that our signature is not driven by one particular group (NAFLD or HC) and reflects true biological signal. Using a forward search we were able to downselect to a parsimonious set of 19 mRNA signature with mean AUROC of 0.98 in discovery and 0.79 in independent validation. Methods for consistent diagnosis of NASH relative to NAFLD are urgently needed. We showed that gene expression data combined with advanced statistical methodology holds the potential to serve basis for development of such diagnostic tests for the unmet clinical need.

## Introduction

Continuous accumulation of fat in hepatocytes leads to Non-Alcoholic Fatty Liver Disease (NAFLD)^1^. While NAFLD is considered a mild condition, Non-Alcoholic Steatohepatitis (NASH), the severe form of the disease, is defined by inflammation and cell damage in addition to the fat accumulation. NASH increases the risk of developing liver fibrosis and may lead to cirrhosis and end-stage liver disease^2–4^. Approximately 25–40% of the general western world population develop NAFLD throughout their lives, of which approximately 30% progress to NASH^5–7^, which is the leading cause for a liver transplant^8,9^ with overall estimated economic burden of $103 billion annually and projected 10-year burden of > $1 trillion in US alone^5,6,10,11^.

Despite truly epidemic proportions, diagnostic options for NASH are very limited^12,13^. Most NAFLD and NASH patients are asymptomatic until relatively late stages of the disease and suspicion of NASH often stems from coincidental findings^2^. Initial screening is based on blood biomarkers such as liver enzymes or insulin resistance, while more complex models also incorporate general risk factors such as age, sex, and BMI; these data are used to calculate risk scores which have reasonable performance at detecting NAFLD but have lower discriminatory power to distinguish NASH^12,14–17^. Based on these scores, a patient is then referred to a hepatologist for further tests involving imaging and/or liver biopsy—an uncomfortable, invasive, expensive, and low throughput procedure which is the gold standard of NASH diagnosis. Yet, even with histological evaluation of liver biopsies, there is poor interobserver and intraobserver agreement further underscoring the complex nature of the disease^18^.

Several studies described differentially expressed genes, microRNAs, and long non-coding RNAs between liver tissues of NAFLD, NASH, healthy, and healthy obese individuals. Few studies also performed meta-analysis of multiple published studies^19–24^. For example, Ryboshapkina and Hammar^24^ used 7 publicly available studies to derive a network of genes related to particular phenotypes and NAFLD progression. However, the results of these independent studies lack consistency, and were not, so far, translated to clinical use. We have previously described the MetaIntegrator framework, which performs a multi-cohort analysis of multiple heterogeneous transcriptome datasets^25–27^. This approach allows us to leverage biological, clinical, and technical heterogeneity to identify robust and generalizable biomarkers that repeatedly validate in independent studies. Our framework is particularly suitable for identifying biomarkers that consistently generalize in diverse contexts, such as real-life patient populations and has been successfully applied to identify novel diagnostic and prognostic markers in cancer, TB, infectious and autoimmune diseases, vaccination, and organ transplant^28–41^.

In this study, we hypothesized that a multi-cohort analysis of transcriptome profiles of liver biopsies from patients with NAFLD or NASH would identify a robust signature for NASH that generalizes across the biological, clinical, and technical heterogeneity of the real-world patient population and will be suitable for clinical development.

## Results

### Data collection and compilation

Our search in GEO and ArrayExpress for transcriptome profiles of liver biopsies from patients with NASH or NAFLD resulted in 12 datasets composed of 812 samples from patients across 7 countries that met our inclusion criteria (Table 1, methods). Overall, the 12 datasets represent a broad spectrum of biological, clinical, and technical heterogeneity. They include samples from adolescents or adults, with or without comorbidities, and profiled using a diverse set of commercial microarrays and RNA sequencing platforms. HCs across these datasets represented real-world heterogeneity as they included those with normal weight, healthy obese, and those suspected of NAFLD. We used 309 samples from 7 datasets identifying differentially expressed genes and 503 samples from 5 datasets for independent validation of those genes. When selecting datasets for discovery and validation, we aimed to maximize the biological, clinical, and technical heterogeneity representation within the discovery datasets, while ensuring that the number of samples allocated to discovery to no more than 50% of the total samples.

**Table 1:** Datasets included in multicohort analysis capture real world heterogeneity.

Dataset ID	Publication			Patient and control populations					Platform
	PMID	Last Author	Year	Country	Age	NASH/NAFLD	HC	Available phenotypes	
Discovery									
E-MEXP-3291	24048683	Cherrington, NJ	2013	US	16–70	N = 16/10 N = 19 Postmortem samples were acquired from NIH-funded Liver Tissue Cell Distribution System. Classification based on presence of inflammation and fibrosis for NASH (regardless of fat deposition), and fat deposition of > 10% for steatosis		Sex, age	GPL6244 Affymetrix Human Gene 1.0 ST Array
GSE126848	30653341	Knop, FK	2019	Denmark		N = 16/15 Histological evaluation of steatosis, activity, and fibrosis (SAF) + Kleiner fibrosis stage^a^	N = 26 Healthy normal weight and overweight individuals, no diabetes or excessive alcohol intake	Sex	GPL18573 RNAseq, Illumina NextSeq 500
GSE33814	23071592	Sültmann, H	2012	Austria	25–78	N = 12/19 Presence of ballooning in combination with variable degree of steatosis and/or inflammation	N = 13 Explant and tumor surgery		GPL6884 Illumina HumanWG-6 v3.0 expression beadchip
GSE37031	23492103	Titos, E	2014	Spain		N = 8/0	N = 7		GPL14877 Affymetrix Human Genome U133 Plus 2.0 Array
GSE63067	25993042	Martínez-Chantar, ML	2015	Spain		N = 9/2 Histology	N = 7		GPL570 Affymetrix Human Genome U133 Plus 2.0 Array
GSE66676	26026390	Inge, TH Teen-Labs Consortium	2015	US	13–20	N = 7/26 NASH Clinical Research Network scoring system^c^	N = 34 Obese, undergoing bariatric surgery, no evidence of steatosis in biopsy	Sex, age, BMI, histology, HDL, LDL, cholesterol, triglycerides	GPL6244 Affymetrix Human Gene 1.0 ST Array
GSE89632	25581263	Allard, JP	2014	Canada	22–68	N = 19/20 Necro-inflammatory Grading System^b^	N = 24 Live donor liver transplant, no steatosis or cirrhosis by imaging or histology	Sex, age, BMI, histology, biochemistry	GPL14951 Illumina expression beadchip
**Validation**									
GSE105127	30297808	Hampe, J	2018	Germany	29–68	N = 5/5 Kleiner NAFLD activity score^a^ (NAS)	N = 9 Scheduled liver resection, exclusion of liver malignancy or bariatric surgery		GPL16791 Illumina HiSeq 2500
GSE130970	31467298	Sanyal, AJ	2019	US		N = 42/30 NASH Clinical Research Network scoring system^c^	N = 6 Live donor liver transplant or patients with ALT fluctuations related biopsy	Sex, age, histology	GPL16791 Illumina HiSeq 2500
GSE48452	23931760	Hempe, J	2013	Germany	38–72	N = 18/14 Kleiner NAFLD activity score^a^ (NAS)	N = 41 Exclusion of liver malignancy during major oncological surgery	Sex, age, BMI, histology, biochemistry	GPL11532 Affymetrix Human Gene 1.1 ST Array
GSE61260	25313081	Hempe, J	2014	Germany	20–86	N = 24/23 Kleiner NAFLD activity score^a^ (NAS)	N = 62 Exclusion of liver malignancy during major oncological surgery	Sex, age, BMI	GPL11532 Affymetrix Human Gene 1.1 ST Array
GSE83452	28679947	Stales, B	2017	Belgium	20–74	N = 126/0 NASH Clinical Research Network scoring system^c^	N = 98 Obese + suspected NAFLD	Sex, age	GPL16686 Affymetrix Human Gene 2.0 ST Array

The 12 datasets included in our analysis span multiple countries, age groups, diagnostic approaches, and technical variation in gene expression platforms. In the NASH/NAFLD and HC columns N indicates the number of samples in the relevant group.

^a^Kleiner DE, Brunt EM 2005.

^b^Brunt EM et al. 1999.

^c^Xnathakos S 2006.

**Figure 1:**
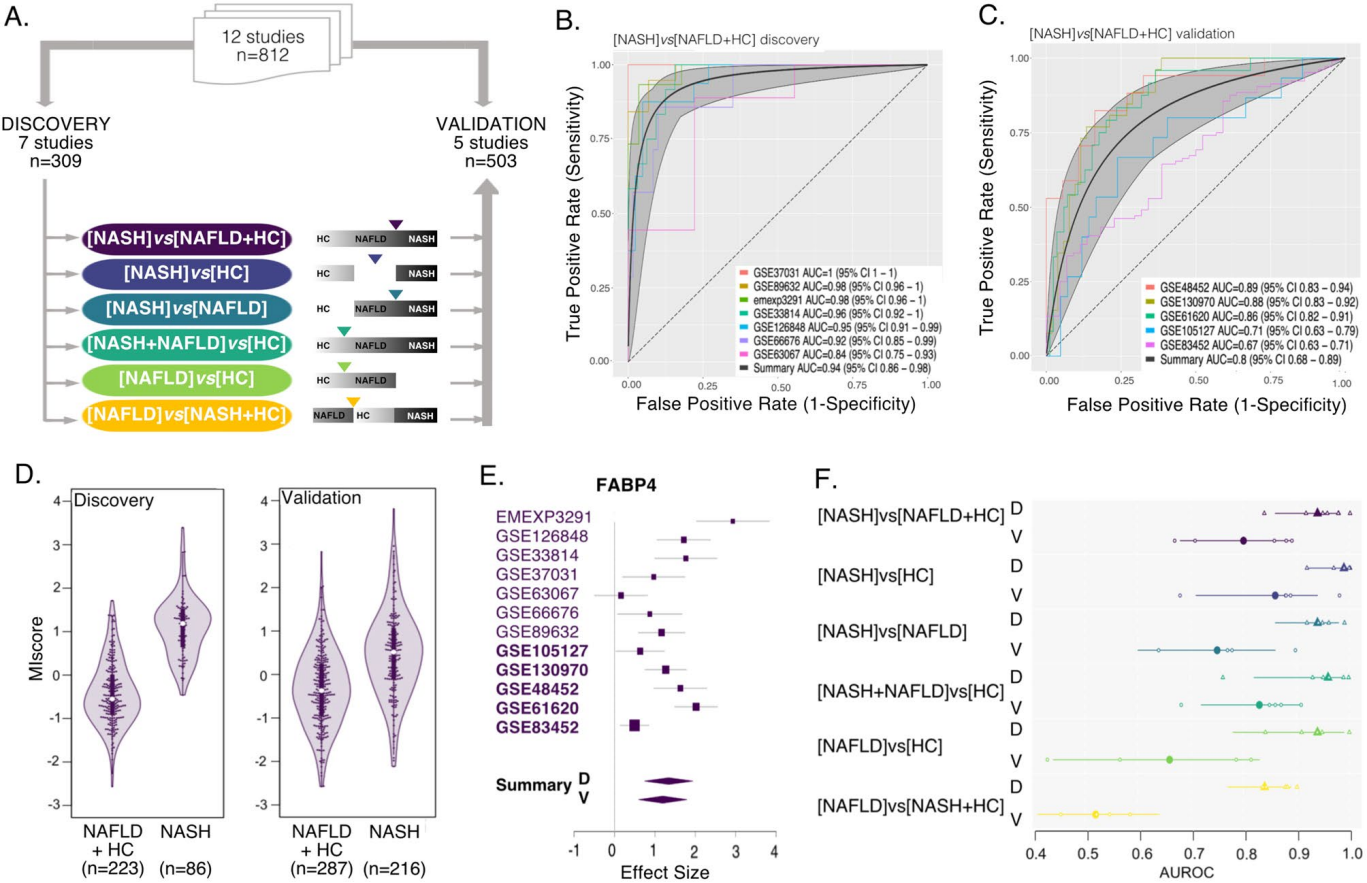
130-mRNA score robustly distinguishes NASH from NAFLD or HC. (**A**) Study design overview. (**B,C**) ROC curves for [NASH]*vs*[NAFLD + HC] signature in (**B**) 7 discovery datasets and (**C**) 5 independent validation datasets. ROCs for individual studies are shown in color, summary ROC is shown in black with 95% confidence interval. (**D**) Violin plot of the [NASH]vs[NAFLD + HC] 130-mRNA score in discovery and validation in each group, n indicates number of samples in each class. (**E**) FABP4 effects sizes across datasets, studies in bold are validation. (**F**) Performance (AUROCs) of all six possible signatures. Summary ROC performance and 95% CI are shown in solid symbol and line, smaller empty symbols show performance in individual studies. Triangles indicate discovery, and circles validation. Color coding same as in (**A**).

We first identified differentially expressed genes in NASH compared to NAFLD and HC groups as our primary comparison. We subsequently extended our analysis to all 6 possible comparisons (Fig. 1A, Table 2). Notably, not all datasets included samples from all phenotypic groups, thus some datasets were excluded from particular comparisons. Membership of datasets in particular comparisons is summarized in Table S1.

**Table 2:** Number of samples used for and genes identified in the six possible gene signatures.

Signature	Class		N samples (% class = 1)		N genes
	1	0	Discovery	Validation	(Up + Down)
[NASH]vs[NAFLD + HC]	NASH	NAFLD + HC	309 (28%)	503 (43%)	130 (85 + 45)
[NASH]vs[HC]	NASH	HC	217 (40%)	431 (50%)	173 (101 + 72)
[NASH]vs[NAFLD]	NASH	NAFLD	160 (44%)	161 (55%)	170 (112 + 58)
[NASH + NAFLD]vs[HC]	NASH + NAFLD	HC	309 (58%)	539 (57%)	50 (34 + 16)
[NAFLD]vs[HC]	NAFLD	HC	206 (44%)	226 (40%)	55 (30 + 25)
[NAFLD]vs[NASH + HC]	NAFLD	NASH + HC	276 (33%)	315 (29%)	41 (20 + 21)

Class indicates the assignment to comparison groups in MetaIntegrator. *HC *healthy control.

### Gene expression signature differentiates NASH from NAFLD or healthy controls

First, we performed a multicohort analysis of NASH vs “others” where others included both the HC and NAFLD groups ([NASH]vs[NAFLD + HC]). At thresholds of |ES|> 0.6 and FDR < 10%, we identified 130 genes that were consistently differentially expressed in NASH vs others (85 over- and 45 under-expressed, Table 2). MetaIntegrator score derived from these 130 genes yielded mean discovery AUROC of 0.94 and validation AUROC of 0.80 (Fig. 1B,C,D), and showed low inter-study heterogeneity of ES for particular genes (e.g. FABP4, Fig. 1E). This strongly suggests that our signature is likely to generalize well in independent datasets, and that a true and robust gene expression signal can be harnessed to reliably distinguish NASH from less severe forms of NAFLD in real world patient populations. 

Despite the encouraging performance of the [NASH]vs[NAFLD + HC] signature, it is possible that wide-spread study design or sample ascertainment methods may have introduced technical variance between the NASH, NAFLD and HC groups, thus artificially inflating any signal in our data. To evaluate this possibility, we explored the performance of all other 5 possible signatures in this data (Fig. 1A,E). For a true biological signal we would expect: (a) signature [NASH]vs[HC] to carry the clearest signal and perform the best; (b) the signature [NAFLD]vs[HC] to carry a weaker signal and perform worse than any signature that involves NASH; and (c) given the progressive nature of the disease [NAFLD]vs[NASH + HC] is unlikely to produce a meaningful signal, serving as an “internal scramble control”. Conversely, if there is a persistent artificial difference between the groups introduced by the sampling process, we would expect that whatever comparison we make would validate similarly well. Indeed, the results from the other 5 signatures (Fig. 1E) support the validity of our analysis. The contrast between two extreme classes in the [NASH]vs[HC] comparison shows the best discriminatory performance with discovery AUROC = 0.99 and validation AUROC = 0.86. The [NAFLD]vs[HC] signature produced an excellent discovery AUC (0.94) but dropped to 0.66 in validation. Lastly, [NAFLD]vs[NASH + HC], which compares the NAFLD group with NASH and HC pooled together as one group, showed discovery performance close to the other five signatures (AUROC = 0.84), but validation performance was essentially random (AUROC = 0.52), as might be expected for this non-useful test case. Altogether, performance of these signatures is consistent with real biological signal as expected. Additionally, since HC is the most distinct group, based on phenotype data we would expect the [NASH + NAFLD]vs[HC] to score high. Indeed, the performance of [NASH + NAFLD]vs[HC] signature is 0.96 in discovery and 0.83 in validation. Notably, [NASH]vs[NAFLD] signature performed similarly to [NASH]vs[NAFLD + HC] (discovery AUROC = 0.94 and validation AUROC = 0.75, Fig. 1F) suggesting that the NAFLD group contributes individually to the overall signal, and [NASH]vs[NAFLD + HC] signature is not driven solely by the HC group.

### Gene expression differences validate known pathways and suggests novel biology

**Figure 2:**
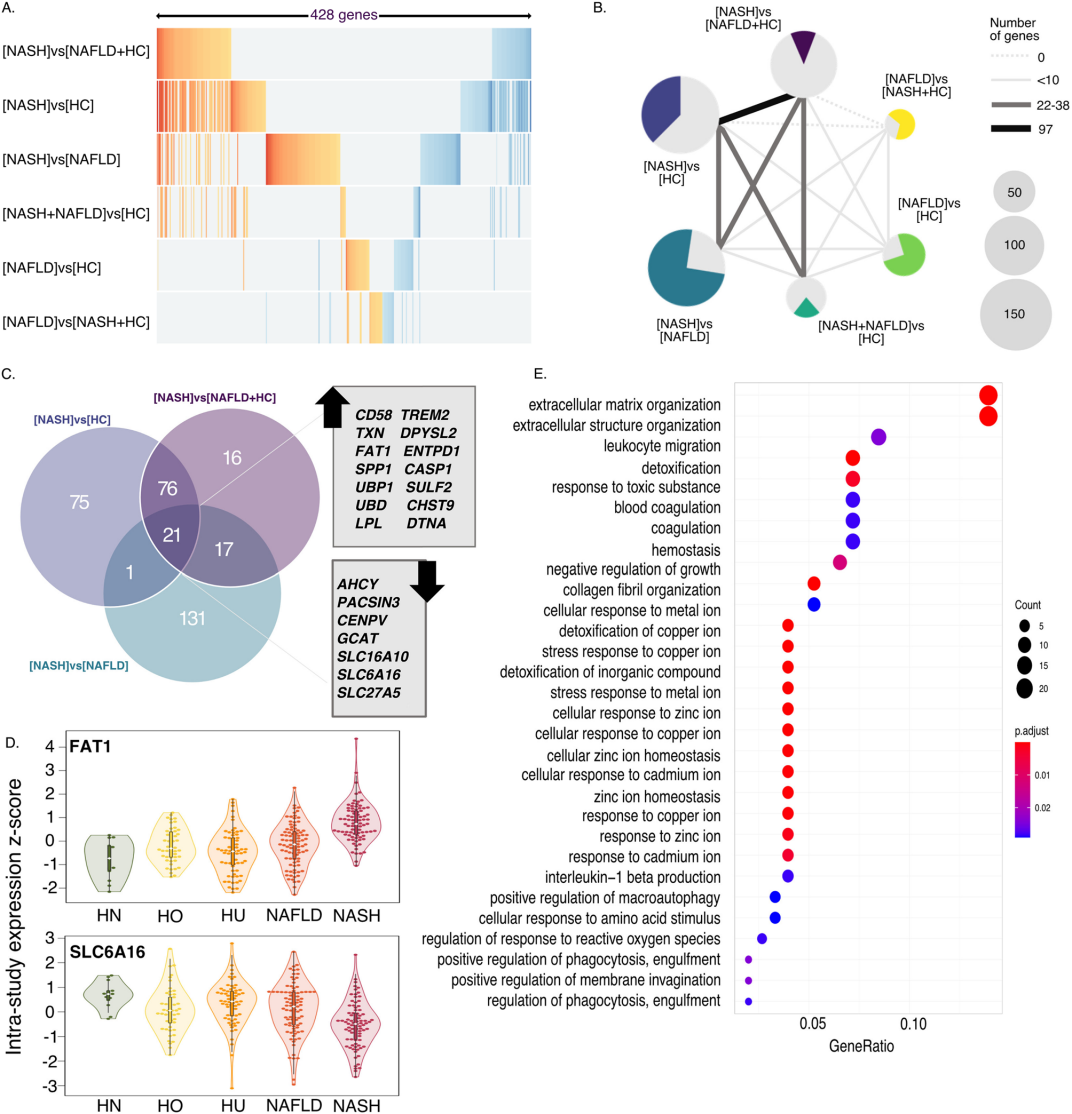
NASH signature gene composition. (**A**) ES of union of all genes (n = 428) in each signature. ES of genes that are not significant for the particular signature were coded as 0 (grey). (**B)** Each signature is represented by a pie chart. Number of genes in each signature is represented by pie size, colored part of the chart represents proportion of genes that are unique to the relevant signature. Thickness of the lines represent number of genes shared by each pair of signatures (as in legend). **(C)** Overlap of [NASH]vs[NAFLD + HC], [NASH]vs[NAFLD] and [NASH]vs[HC] signatures. Genes common to all three signatures (n = 21) are listed according to their direction of change (up or down) in [NASH]vs[NAFLD + HC] signature. (**D**) Violin plots of zscores of expression of 2 representative genes (FAT1 for over-expressed and SLC6A16 for under-expressed) listed in C. Groups are color coded based on classification (HN = healthy normal BMI, HO = healthy obese, HU = healthy unknown BMI, NAFLD and NASH). In analysis all healthy were considered as one group, regardless of BMI status. (**E)** Pathway enrichment analysis of [NASH]vs[NAFLD] signature.

To gain biological insight we compared the differentially expressed genes between the signatures. Each signature has between 41 and 173 differentially expressed genes (Table 2) and altogether the six signatures encompass 428 genes (Table S1). The four signatures that include NASH were consistent with each other. For example, effect sizes (ESs) for genes captured in those four contrasts maintain their directionality (Fig. 2A) and the four signatures that include NASH patients as cases share a substantial number of genes among them (Fig. 2A,B). This is in contrast to the weaker [NAFLD]vs[HC] and [NAFLD]vs[NASH + HC] signatures for which majority of the genes were only detected in that particular comparison and not shared by any other signature (Fig. 2B).

We then focused on the genes shared between the three signatures that distinguish NASH as a separate group: [NASH]vs[NAFLD + HC], [NASH]vs[HC], and [NASH]vs[NAFLD] (Fig. 2C). This comparison also underscores the individual contributions of HC and NAFLD groups to the [NASH]vs[NAFLD + HC], suggesting that the overall signature is not driven by either on group. There are 14 upregulated and 7 downregulated genes that overlap between the three signatures (Fig. 2C,D). Notably, 13 of the 21 genes were previously implicated in hepatocellular carcinoma (HCC) progression or survival—11 of which are upregulated genes and 2 downregulated. Similarly to our results, the human protein pathology atlas^42,43^ (a database of protein levels measured in human samples) showed higher levels of *CD58, UBP1, TREM2*, and *SPP1* are significantly associated with poorer survival in HCC patients while higher level of *SLC27A5* is associated with favorable prognosis. Also, increased mRNA expression of *TXN, SULF2, CASP1, LPL, ENTPD1, SPP1, DTNA*, and *UBD* was associated with poor HCC prognosis, while mRNA expression of *ACHY* and *SLC27A5* was potentially protective for HCC patients^44–54^. These associations are in complete concordance with our results, suggesting that there is already a significant induction of the oncogenic pathways during NASH. Finally, several genes in the [NASH]vs[NAFLD + HC] signature (e.g. CHST9, DPYSL2) have not been previously implicated in liver disease and could potentially provide novel biological insights into NASH etiology.

We carried out gene set enrichment analysis on gene groups for all six signatures. Interestingly, only [NASH]vs[NAFLD] signature (Fig. 2E), which also contains the largest number of unique genes (131), yielded significant enrichment results. The top pathways significantly over-represented in [NASH]vs[NAFLD] are extracellular matrix organization, leukocyte migration, and detoxification (Fig. 2E) which is consistent with the onset of hepatic inflammation as a hallmark of NASH versus NAFLD.

### [NASH]vs[NAFLD + HC] optimization identifies small set of genes that retain the performance of the signature

**Figure 3:**
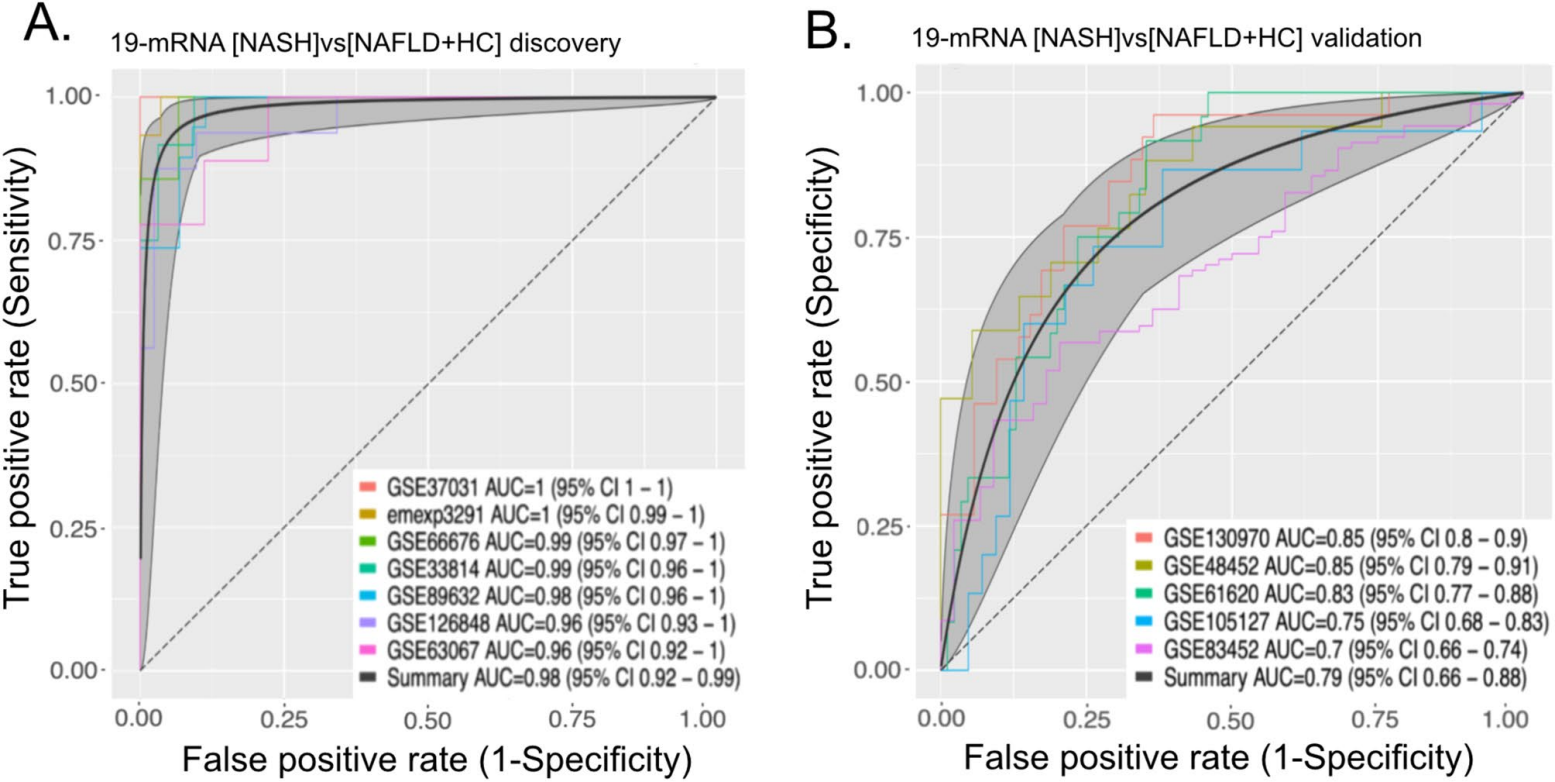
A parsimonious set of 19 genes retains the full performance of [NASH]vs[NAFLD + HC] signature. (**A**) Performance of 19-gene signature in discovery studies. (**B)** Performance of 19-gene signature in validation studies.

While [NASH]vs[NAFLD + HC] carries significant signal to discriminate between NASH from NAFLD or HCs, measuring expression of 130 genes in clinical setting might be unpractical and unnecessary. In addition, while all the genes we used are differentially expressed in the NASH group, considerable correlation between the expression of some signature genes renders their contribution to the performance of the final score redundant. We used a greedy forward search to narrow down the number of genes necessary for our signature performance. We were able to minimize our gene set to 19 genes (Table S1) while optimizing for discriminatory performance between NASH and NAFLD or HC groups in the 7 studies used for discovery. We then re-computed a diagnostic score using only those 19 genes, and show that the 19 gene score performs as well as the complete list (validation summary AUROC 0.79 vs summary AUROC 0.80; Fig. 3A,B).

## Discussion

To identify a persistent and reproducible gene expression signature for NASH, we performed the most comprehensive meta-analysis of published liver gene expression to date, encompassing 12 datasets (812 samples) that represent real world patient populations. We successfully leveraged both biological and technical heterogeneity in identifying a gene expression signature that robustly distinguishes NASH from NAFLD or HCs.

Multiple tests and scoring systems have been developed for non-invasive diagnostics for NAFLD and NASH. These perform well enough for detecting NAFLD, but not NASH, or are limited to particular populations^12,16^. Our 19-gene signature achieved a performance of mean AUROC = 0.98 in discovery and AUROC = 0.79 in independent, blind validation in 5 datasets that included multiple populations or phenotypes. This strongly suggests that our approach has great potential for development of gene-expression-based diagnostic test for NASH. Gene enrichment analysis of the NASH signature reflects the diagnostic hallmark of NASH, relative to NAFLD—inflammation. Interestingly, many of the genes we identify were previously implicated as potential markers for HCC survival and progression. This result suggests that the molecular processes involved in progression of NASH to cirrhosis and HCC are evident in at least some NASH patients and could serve as basis for development of endotyping strategy for NASH.

As part of this study, we also examined all six possible signatures, and show that the discriminatory performance of [NASH]vs[NAFLD + HC] signature is unlikely to stem from any technical variation introduced through general study design or sample ascertainment process. Furthermore, we investigated whether [NASH]vs[NAFLD + HC] performance is driven by the HC group alone or reflects true signal, discriminating NASH from both HC and NAFLD. To this end, we compared both the performance and composition of the three signatures: [NASH]vs[NAFLD + HC], [NASH]vs[NAFLD] and [NASH]vs[HC]. If [NASH]vs[NAFLD + HC] is driven only by the HC group we would expect the [NASH]vs[NAFLD] signature to perform poorly and that the gene composition of [NASH]vs[NAFLD + HC] would be essentially a subset of the [NASH]vs[HC]. On the other hand, if both groups contribute to the signature, we should see a similar performance and significant overlap in composition between [NASH]vs[NAFLD + HC] and either of the other two signatures. Our results clearly suggest that [NASH]vs[NAFLD + HC] signature is driven by both of HC and NAFLD groups.

Ultimately, not all six signatures perform equally well in our analysis. It is clear that the distinction between NAFLD and HC ([NAFLD]vs[HC]) proved to be more difficult than distinguishing NASH from any combination of HC and NAFLD. This is expected as it mirrors the situation in the clinic. Also, the large spectrum of HC samples in the 12 datasets analyzed here (Table 1) suggests a significant overlap between definitions of NAFLD, healthy obese, and healthy. For example, HC samples in GSE66676 were taken from obese patients undergoing bariatric surgery. We believe that this wide variation in control population, at least in part, explains the relatively large variation in our validation performance. In particular, GSE83452, the worst performing validation dataset, is the only validation study that does not include patients diagnosed as NAFLD and uses obese as control population. The control samples in this dataset were taken from people who had abnormal liver enzymes and had a biopsy taken, which was declared not NAFLD by a pathologist. Thus, in our opinion, the relatively large drop in performance between the discovery and validation is at least in part due to the differences in diagnostic approach and potentially inter-individual differences in histological evaluations between different pathologists^18,55^. This variability further underscores the need for more cohesive and objective diagnostic framework for NASH.

 There are some limitations to our study. First, while our approach accounts for biological, technical, and clinical heterogeneity, it is limited by the variability captured in the datasets available to us. It is possible that incorporation of additional sources of variability—such as age, ethnicities, geographical areas, or technical platforms, would lead to reduced performance in these settings. Thus, further retrospective validation in independent cohorts is needed to ensure that we can refine our gene signature and achieve a level of performance that has clinical utility. Yet this also highlights the strength of our framework: as new datasets become available, they can and should be incorporated in the analysis producing updated and refined signature models. Second, to reach clinical utility, our 19-gene signature should undergo further algorithmic refinement. Notwithstanding, the computational refinement process can also be computationally expensive, presents unique challenges and ideally should be done when more data is available. Thus, we believe that this type of signature optimization is beyond the scope of this manuscript. Third, the holy grail of NASH diagnostic is non-invasive molecular test that would not require liver biopsy. Our analysis indicates that the [NASH]vs[NAFLD + HC] signature is driven by changes in composition of immune cells (e.g. leukocyte migration), and we expect to be able to detect such processes in blood as well. Our experience with other diseases^28,33,40,41,56–58^ suggests that given enough data, the MetaIntegrator framework would be successful in developing blood based signature. However, transcriptomic blood data for NASH patients is very sparce. Hence, our strategy is first to identify and validate NASH gene signature based on liver data and translate it into blood, when blood transcriptomic data becomes available. Finally, in this work we focused on feature selection which is only a first step in building a model. Our score is a difference of geometric means, it does not produce probability score and therefore some calibration metrics, such as calibration curves or Hosmer–Lemeshow test are not applicable. Rather, these metrics if applicable, should be included in final model evaluation.

Taken together, we believe that our work provides a solid foundation for development of gene-expression-based test for NASH. Accurate liver gene expression testing could help with NASH diagnosis, patient care, and potentially drug development. For example, it could inform retrospective analysis of clinical trials of failed NASH therapies—potentially opening an avenue for more successful characterization of patient subgroups that benefited from the treatment and repositioning of otherwise failed drug candidates. We envision that, in addition to the current standards of histological evaluation by qualified pathologists, a gene-expression-based NASH diagnostic will add value in clinical decision making and promote standardization in the field. Biopsies represent only a small area of the liver, therefore histological changes that have not widely spread could be missed. In principle, a gene-expression-based diagnosis would have the added value of reflecting changes in cellular microenvironment that occur outside of that particular area.

To summarize, our results demonstrate that gene expression analysis harbors an exciting opportunity for development of diagnostic test for NASH. We believe that this work provides a solid foundation for further development, both in terms of algorithmic refinement of the presented signatures and addition of other datasets that would help develop accessible, high throughput and reliable diagnostic. With further prospective validation, our results hold the potential for breakthrough diagnostic test for NASH.

## Methods

### Data collection

We searched public gene expression repositories (GEO^59^ and ArrayExpress^60^) for datasets that included transcriptome profiles of liver biopsies from patients with NASH in January 2020. We identified 83 datasets with 3,359 samples. We excluded datasets that did not meet the following criteria: human, liver tissue, includes at least 5 patients in either NAFLD or HC groups and at least 5 patients in the NASH group. Twelve datasets met the inclusion criteria (Table 1). We then manually curated the 12 datasets to ensure integrity of phenotypic data, diagnostic criteria of NASH, NAFLD, and HC patients, and for general match between the deposited data and the numbers cited in associated publication when available.

### Data preprocessing

For each dataset, we downloaded raw expression data and pre-processed using standard methods. Specifically, we applied RMA to all data from Affymetrix platforms^61,62^ and used *limma* package^63,64^ with quantile normalization for Illumina and other commercial arrays. For RNAseq datasets, we downloaded the associated SRA read fastq files and used FastQC for initial quality control. We then used STAR v2.2^65,66^, human genome GRCh38^67^ and GENCODE^68,69^ v32 human genome annotation for read alignment and gene expression quantification, as previously described^70^. To facilitate integrated analysis, we used the Annotation Dbi and Hs.org^71^ packages to map probe and gene identifiers in each dataset to Entrez Gene identifiers (IDs). We used sample identifier to match phenotypic data from the databases to expression data and used the phenotypic and expression data to ensure sample uniqueness. We found no duplicated samples between the datasets.

### Multicohort analysis

We used the R package MetaIntegrator for multi-cohort analysis^26,27,72^. Briefly, to identify robust changes in gene expression, in each discovery dataset MetaIntegrator calculates gene specific Hedges’ g effect size (ES) between two classes labelled as 1 and 0, as well as Benjamini–Hochberg False Discovery Rate adjusted p-value for that ES. The pooled ES of each gene, across all datasets, is computed using DerSimonian & Laird random-effects model and Fisher’s sum of logs method is used to summarize p-value of ES across datasets.

We a priori divided the datasets into two groups: (1) 7 datasets (309 samples) as “discovery cohorts” and (2) 5 datasets (503 samples) held out as independent “validation cohort” (Table 1). When dividing the studies into discovery and validation we sought to maximize the technical and biological heterogeneity encompassed by the discovery studies, while keeping the number of samples for discovery bellow 50%. To avoid overfitting we also designate all studies from the same research group either for discovery or validation. For each comparison, we defined the more severe phenotypic group as “case” and the less severe group as “control” (Table 2). We used the more stringent Leave-One-Study-Out (LOSO) cross-validation within the discovery cohorts to identify differentially expressed genes, whereby each of the discovery datasets is left out in a round-robin fashion to obtain ES for each gene. In each iteration, we applied the ES and q-value thresholds, and selected the genes that met these thresholds in every iteration. This ensures that gene’s ES is not driven by one particular dataset and results in a list of genes that are consistently positively or negatively differentially expressed between the classes. We examined the gene lists produced by MetaIntegrator *filterGenes* function over multiple ES and q-value thresholds (0.6–0.8, and 0.1–0.01 respectively), adopting the cutoffs of |ES|> = 0.6 and FDR p < 0.1. Notably, the FDR in this case refers to the significance level associated with the pooled effect size, not to a separate testing of differential expression. These thresholds were determined based on the guideline from power estimate (Figure S1) as well as to allow for meaningful pathway analysis in all 6 signatures. Expression of the selected genes is then combined into a score:

$$MIscore = zscore\left( {Geometric\; Mean\left( {positive\; genes} \right) - Geometric\; Mean\left( {negative\; genes} \right)} \right)$$

### Gene set enrichment analysis

We used *enrichGO* function from the R package *clusterProfiler* to perform gene enrichment analysis^73–75^. The package supports overrepresentation test against the entirety of organism specific GO annotation as represented in the OrgDb object, and provides Benjamini–Hochberg adjusted p-value for the observed overrepresentations. We used union of all genes expressed in the discovery datasets, for general background.

### Approval for human experiments

All studies included in this meta-analysis obtained informed consent of the human subjects and were performed in accordance with relevant named guidelines and regulations governing the study. No new subjects were recruited for the purpose of this work.

## Supplementary Information

Supplementary Information 1.

Supplementary Information 2.
