# Supplementary material for: A robust gene expression signature for NASH in liver expression data
Source: Sci Rep. 2022 Feb 16;12:2571. doi: 10.1038/s41598-022-06512-0 (PMC8850484; doi:10.1038/s41598-022-06512-0)
Supplement: Supplementary file 1 — Supplementary Information 1. [file 41598_2022_6512_MOESM1_ESM.docx]

**­A robust gene expression signature for NASH in liver expression data**

Yehudit Hasin-Brumshtein^1^, Suraj Sakaram^1^, Purvesh Khatri^2,3^, Yudong D. He^1, +^, Timothy E. Sweeney^1, +^

^1^Inflammatix, Inc., 863 Mitten Rd, Suite 104, Burlingame, CA 94010, USA

^2^Institute for Immunity, Transplantation and Infection, School of Medicine, Stanford University, Palo Alto, CA 94305, USA

^3^Center for Biomedical Informatics Research, Department of Medicine, Stanford University, Stanford, CA 94305, USA

^+^ Corresponding author:

Timothy E Sweeney ([tsweeney@inflammatix.com](mailto:tsweeney@inflammatix.com)) or Yudong D He ([yhe@inflammatix.com](mailto:yhe@inflammatix.com))

**Supplementary figure and table:**

**Figure S1 – power analysis for NASH**

**Figure S1: Power estimation for [NASH]vs[NAFLD+HC]**

Power was estimated using *calcMetaPower* function from the MetaIntegrator package, for three possible levels of heterogeneity (low, moderate and high). Vertical line indicates ES=0.6, and horizontal power=0.8.

**Supplementary Table 1:**

Effect sizes of 428 union genes identified in all 6 signatures. Numbers are effect size in that comparison, while NA indicates that the gene is not part of that signature. Supplied as separate excel file.
